# Failure of Development of the Corpus Callosum

**Published:** 1911-11

**Authors:** 


### Failure of Development of the Corpus Callosum

La Salle Archambault fills the entire original department of the *Albany Medical Annals* for September, 1911, with an exhaustive treatise on Agenesia of the Corpus Callosum, illustrated with four full-page half tones and six cuts. His conclusions are as follows:

1. In cases of so-called agenesia of the corpus callosum, we have to do merely with an absence of the callosal commissure proper.

2. In a considerable number of cases, foetal hydrocephalus is the underlying cause of this anomaly. This lesion may act either by destroying a large part of the periventricular callosal layer, or by determining ventricular symphysis which then interferes mechanically with the development of the callosal commissure.

3. The occipito-frontal fasciculus simply represents, as Sachs originally maintained, a heterotopy of the corpus callosum. It does not exist in the normal cerebrum.

4. The fornix is formed not only by fibres derived from the gyrus hippocampi but also by fibres which originate in the gyrus fornicatus and reach it by traversing the corpus callosum.

5. The internal sagittal layer of the fronto-parietal lobe, the reticular zone of Sachs, represents the corona radiata of the gyrus fornicatus or first limbic convolution.

6. The anterior commissure probably enters into the constitution of the temporal segment of the tapetum. Aside from this possible element, the tapetum is formed exclusively by the fibres of the corpus callosum.

